# A Study on the Effects of Hypothyroidism on the Senses: A Comprehensive Narrative Review

**DOI:** 10.7759/cureus.65684

**Published:** 2024-07-29

**Authors:** Eric LeVasseur, Maiah Fogel, Deepesh Khanna

**Affiliations:** 1 Foundational Sciences, Nova Southeastern University Dr. Kiran C. Patel College of Osteopathic Medicine, Clearwater, USA; 2 Foundational Sciences, Nova Southeastern University Dr. Kiran C. Patel College of Osteopathic Medicine, Fort Lauderdale, USA

**Keywords:** graves' ophthalmopathy, tsh, levothyroxine, thermoregulation, vision, hearing, taste sensation, smell, hypothyroidism on senses, hypothyroidism

## Abstract

Hypothyroidism has been found to have long-term effects on each of the senses, but with proper treatment, many of them can be significantly minimized. This paper analyzes the research on the impact of hypothyroidism on the senses of smell, taste, hearing, vision, and thermoregulation. Data were collected from the National Library of Medicine, PubMed, and Google Scholar databases using the keywords “hypothyroidism,” “taste,” “smell,” “vision,” “hearing,” and “thermoregulation.” Approximately 413 articles were found when searching with these parameters, and 30 were used in this paper. Studies were excluded if they were outside this paper's scope or older than 2012. Studies were included if they specifically focused on hypothyroidism and one of the five listed senses. Patients with hypothyroidism had a significantly increased risk of sensorineural hearing loss, decreased perception of the blue-yellow color axis, decreased sense of olfaction and number of olfactory bulbs, and decreased thermogenesis. Hypothyroidism was also found to show increased length of COVID-19-induced anosmia and decreased bitter taste perception. It can be concluded that hypothyroidism has many effects on the senses, particularly an increased risk of sensorineural hearing loss. More studies need to be done on these effects.

## Introduction and background

Hypothyroidism has been a well-studied condition over the past few decades. It is a condition that affects nearly 400,000 people globally, with another 400,000 presumed to be undiagnosed [1]. Hypothyroidism is also more prevalent in women, people over 65 years of age, and White individuals [2]. The causes of hypothyroidism are varied as well, making it difficult to know exactly what caused it. Areas of the world with iodine deficiency are more likely to have individuals with hypothyroidism due to iodine’s role in thyroid hormone synthesis [2]. Even areas that are normally iodine-sufficient can affect patient populations, such as pregnant women, and increase the prevalence of hypothyroidism [2]. Autoimmunity also plays a big role, especially in areas that are iodine-sufficient. The most common cause of hypothyroidism is autoimmune thyroiditis, where the thyroid tissue antigen-presenting cells recognize self-antigens and present them to T-cells. This leads to autoantibody production and degradation of the thyroglobulin and thyroid peroxidase, causing decreased production of necessary thyroid hormones [1]. Genetics also play a role but are not as well understood as the other causes. Genes related to autoimmunity and thyroid-stimulating hormone (TSH) concentrations are the main subjects when looking into hereditary hypothyroidism. It is estimated that the heritability of TSH is about 65% from parent to offspring, so there is an increased risk of a parent with hypothyroidism passing it down to their children [2].

The main symptoms of hypothyroidism are weight gain, fatigue, difficulty with concentration, and depression [1]. These symptoms are managed using a thyroid hormone known as levothyroxine, which is used to replace the missing thyroid hormone in patients. While the above-mentioned symptoms are well known and managed through medication, new research is showing more how hypothyroidism is also linked to our senses of smell, taste, sight, hearing, and thermoregulation. For instance, the effects on smell and taste were two of the first research regarding hypothyroidism in 1975 [3]. In untreated hypothyroidism, 39% of patients had noticed a decreased sense of smell [3]. In a similar study, it was found that about 5% of patients with hypothyroidism, even after being treated with levothyroxine, were still experiencing dysgeusia [3]. A study on hypothyroidism and vision linked a vision-threatening disease known as Graves' ophthalmopathy in rare cases of hypothyroidism [4]. Graves' ophthalmopathy causes periorbital swelling and elevated thyroid-stimulating immunoglobulin in the body, and if the swelling is left untreated, it will lead to permanent loss of vision [4]. Graves' ophthalmopathy occurs in about 2%-7.5% of all patients with hypothyroidism, and while rare, that is what makes it even more important to be screened for early as it can be prevented with the use of steroid treatment [4]. This is just a small fraction of the effects on the senses that hypothyroidism has. This review will continue to analyze the current research surrounding hypothyroidism’s effect on the senses and how this may be an understudied field in a complex medical condition.

## Review

Methods

**Figure 1:**
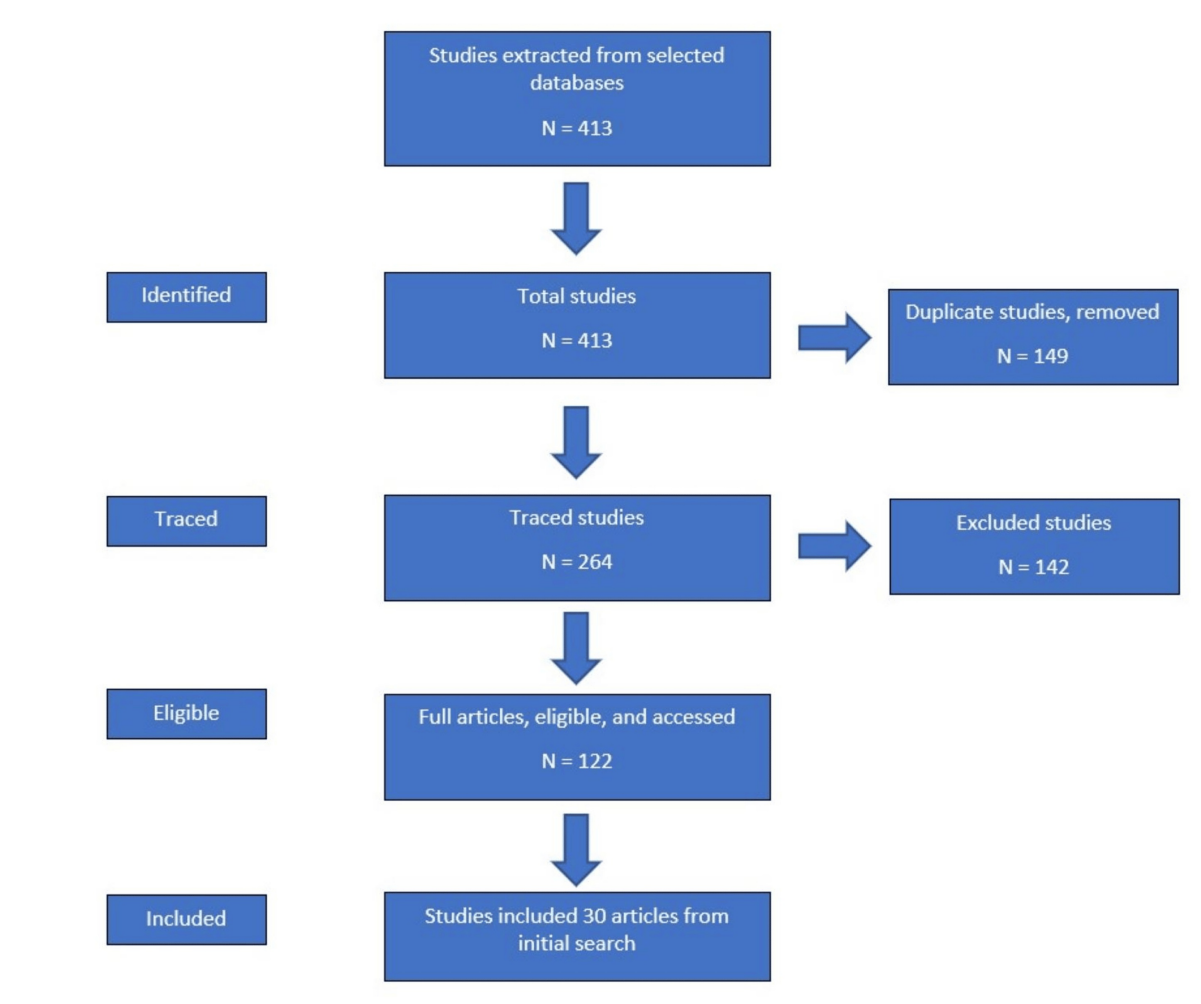
Study selection flowchart

To conduct this narrative review, a search into the National Library of Medicine, PubMed, and Google Scholar databases was performed, looking for articles with the keywords “hypothyroidism,” “taste,” “smell,” “vision,” “hearing,” "dysgeusia," "anosmia," and “thermoregulation.” Approximately 128 articles were found when searching with these parameters. Studies were excluded if they were outside the scope of this paper and published before the year 2010. Studies were then included if they specifically focused on hypothyroidism and one of the five listed senses of the human. The articles gathered were focused on different types of hypothyroidism and varying patient populations. Some articles focused on two of the above senses and were able to provide data over both sets of parameters. An equal number of articles were pulled for each sense to give a representative analysis for this review. A total of 48 articles were found to be eligible for this review. However, 30 articles were used for the final analysis, as shown in Figure 1.

Discussion

Each of the below sections covers one area of sensation and the effect of hypothyroidism on it. Some of the senses are more thoroughly researched than others, which is also a major point of disparity that this paper will try to address.

Effects on Smell

Thyroid hormones are crucial to the body’s ability for growth and cell differentiation, yet with an important sense like olfaction, there are very few studies of hypothyroidism’s effects on it. A 2015 study took 45 patients aged between 18 and 60 diagnosed with hypothyroidism and compared their sense of olfaction to a 45-person control group [5]. These participants were tested on parameters including odor threshold, identification, and discrimination of the smell, with the hypothyroid group scoring significantly lower than the control group in all three categories [5]. When comparing the results with the different levels of TSH, free triiodothyronine (FT3), and free thyroxin, only the FT3 levels showed a significant correlation, suggesting that only FT3 influences olfaction [5].

Another study comparing a group of 21 individuals with hypothyroidism and 31 controls showed no significant differences in perception of smell [6]. However, this study further separated its experimental group participants into subclinical and clinical hypothyroidism individuals. Subclinical hypothyroid individuals were determined to have less severe symptoms and more normalized levels of TSH than their clinical hypothyroid counterparts. Individuals who were determined to have clinical hypothyroidism were found to have significant differences in levels of olfactory stimulation of cranial nerves 1 and 5 between both the subclinical hypothyroidism individuals and the control group [6]. This posits an interesting point suggesting that as one’s hypothyroidism worsens or is left untreated, the sense of olfaction will continue to deteriorate.

Subsequent conditions that arise from hypothyroidism can also affect olfaction as well. A study was conducted taking 68 patients with hypothyroidism and subsequent Hashimoto’s thyroiditis to compare their olfaction ability with 66 control patients [7]. The focus of this study was to find out the volume of olfactory bulbs within each patient population, the function of which is to take up olfactory stimuli, process them, and send them to the brain [7]. It was found that right and left olfactory bulb volumes were significantly less in patients with hypothyroid Hashimoto’s thyroiditis than patients with normal thyroid and even patients with euthyroid Hashimoto’s thyroiditis, leading to the belief that the main cause of decreased olfactory bulb volume is due to hypothyroidism [7].

Tying this research into more current medicine, a study was also done with a group of 12 hypothyroid patients and 24 controls after COVID-19 infection to test post-COVID-19-induced anosmia [8]. All participants had similar demographics and severity of COVID-19 infection, and it was found that patients with hypothyroidism were significantly more likely to have a prolonged duration of approximately 10-15 days of post-COVID-19-induced anosmia [8].

One of the glaring issues of the above studies is how small the sample sizes are. However, with the data being as important as it is, it further stresses how more research needs to be conducted with larger groups to confirm the above results. This is especially important regarding the second study discussed, as further testing could reveal olfactory stimulation as a marker for the more severe form of hypothyroidism and help with providing necessary treatment for patients. It is also important to note that while decreased olfaction is found in a considerable number of patients with hypothyroidism, it is very rarely talked about and is treated as an insignificant finding concerning other symptoms. However, the implication of decreased smell can negatively impact the patient's day-to-day life and should be treated with more care than it currently is.

Effects on Taste

Taste is another sense that is altered with hypothyroidism. About 50% of all patients experience some form of dysgeusia or altered sense of taste, typically making foods taste more bitter or metallic [9]. However, this symptom is typically not focused on when discussing hypothyroidism and is often overlooked by individuals as stemming from a thyroid hormone deficiency. A study conducted with 32 individuals with untreated hypothyroidism and 31 controls sought to test the relationship between sour, sweet, salty, and bitter tastes between the two populations [10]. It was found that bitter and sweet tastes were significantly diminished in patients with hypothyroidism compared to the control group [10]. Participants in the uncontrolled hypothyroidism group were then started on L-thyroxine for three to six months. After this period, testing showed a significant improvement in the participant's ability to recognize sour, sweet, salty, and bitter tastes [10].

A similar study was conducted with 28 subclinical hypothyroidism patients and 31 controls, which yielded similar results [11]. This study also showed significantly diminished bitter and sweet tastes that significantly improved after treatment with L-thyroxine [11]. Bitter taste was found to be the most significantly affected and was compared with levels of different thyroid hormones within the participants [11]. It was found that FT3 was positively correlated with bitter taste after patient treatment with L-thyroxine [11].

L-thyroxine itself has been studied closely with the bitter taste receptor TASTR2 [12]. A study on these receptors using human and mouse thyrocytes showed that agonists of TASTR2 receptors would inhibit the concentrations of TSH-dependent calcium ions [13]. These agonists also reduced iodide efflux, which in turn led to a reduced level of circulating thyroid hormones such as T3 and T4 [13]. A more specific bitter taste gene has been identified known as TAS2R38, which is being used to help diagnose thyroid dysfunction [14]. This gene is being found in patients with either hyper- or hypothyroidism and is caused by single nucleotide polymorphisms [14].

These studies are specifically linked and show the important connection between taste receptors and thyroid hormones. Upon treatment of undiagnosed hypothyroidism, there is a significant improvement in bitter taste through the TASTR2 receptor, which, in turn, leads to increased production of thyroid hormones essential to proper thyroid functioning. This research is paramount in assessing dysgeusia as a marker for untreated hypothyroidism.

Effects on Hearing

Hearing impairment is the most researched impairment of the senses regarding hypothyroidism. This impairment notably results from changes in the structure of the tectorial and basilar membranes of the inner ear [15]. A study of 41 participants with primary hypothyroidism secondary to Hashimoto’s thyroiditis and 20 controls were subjected to hearing threshold tests ranging between 250 and 8,000 Hz [15]. Participants were also aged between 16 and 50 to avoid any potential conflict with age-related hearing changes [15]. Sensorineural hearing loss was found in 16 of 41 of the hypothyroid group, typically at frequencies of 250, 500, and 6,000 Hz [15]. Whereas in the control group, only 1 of 20 controls were found to have some form of sensorineural hearing loss [15].

However, this is not just a concern for adults and the elderly diagnosed later in life. A lot of current research is also observing these audiological changes in children with congenital hypothyroidism as well. A study of 32 children with congenital hypothyroidism and a median age of about 15.2 were tested for their ability to hear eight different auditory stimuli at eight different frequencies [16]. Hearing loss was denoted as mild if it was between 20 and 40 db hearing level (HL), and severe if it was greater than 40 db HL [16]. A marked disparity in hearing impairment was observed between children diagnosed with congenital hypothyroidism and their healthy counterparts, with the impairment intensifying at elevated frequencies. Notably, even with prompt and appropriate treatment initiated prenatally for congenital hypothyroidism, affected children were still found to have varying degrees of hearing impairment by age 15. Furthermore, the degree of hearing loss was directly correlated with the severity of the congenital hypothyroidism [16].

Another study of 66 individuals aged 3-21 with congenital hypothyroidism found that 19 individuals had some form of hearing impairment and that five of the 19 had severe hearing impairment [17]. Another study of 112 individuals aged 5-16 found that diagnosis of congenital hypothyroidism also increased the risk of developing central auditory processing disorder [18]. In a meta-analysis of 23 studies involving 2,183 children with congenital hypothyroidism and 862 control children, it was observed that the risk of developing some form of hearing impairment was 9.2% in children with congenital hypothyroidism, compared to 2.3% in those without the condition [19].

Even on the cellular level, researchers have noticed changes in certain transcription factors can drastically alter the hearing impairment of a fetus with congenital hypothyroidism [20]. A study on mice analyzed two very similar pituitary transcription factors, PROP1 and POU1F1, which, when mutated, cause the inability to synthesize thyroid hormones [20]. However, these two factors have significantly different effects on hearing. Mutation in PROP1 leads to offspring with mild hearing deficiency from 6 to 12 weeks of age, whereupon normal hearing is reestablished [20]. However, a mutation in POU1F1 is much more severe and leads to permanent deafness [20]. Congenital hypothyroidism is a condition that affects 1 in 3,500 newborns. Hence, isolating a particular transcription factor that can cause so much damage is important for future research.

Effects on Vision

Vision was found to be one of the senses least researched in terms of hypothyroidism. This was quite shocking as hypothyroidism causes a notably increased risk of age-related macular degeneration and affects the development of cones [21].

Hypothyroidism also affects the development of the hippocampus and decreases cerebral blood flow, which can lead to changes in one’s visuospatial processing [21].

Thankfully, some parameters of vision appear to be unaffected by hypothyroidism. A study on six hypothyroid participants and six controls sought to test whether hypothyroidism affected one's speed of visual perception [22]. The study utilized the flicker fusion frequency test in which light is flashed at increasing frequencies until it is perceived to be continuous. The lowest frequency that a participant notes the light is continuous is termed the flicker fusion frequency. In this study, there was no significant difference between the scores of the hypothyroid participants and the control group [22].

Research has also been done on the effects of hypothyroidism on color perception. A total of 38 hypothyroid patients were compared to 20 euthyroid patients to determine the effect of hypothyroidism on color contrast sensitivity [23]. Thyroid hormone itself is important for the eye as it helps with repressing short-wave opsin (important for blue color perception) and expressing medium-wave opsin (important for green color perception) [23]. Initially, the study examined 38 hypothyroid patients who had not received treatment and compared their clinical measurements to those of patients with normal thyroid function (euthyroid). Subsequently, these patients were reassessed after a median period of 90 days following treatment. The posttreatment results were then compared both to their pretreatment values and to the metrics of the euthyroid group, as documented in [23]. It was found that when going from untreated to treated hypothyroidism, these patients’ red-green color contrast sensitivity improved to the levels of their euthyroid counterparts [23]. However, both the untreated and treated hypothyroid patients still had a significant decrease in their blue-yellow color contrast sensitivity compared to the euthyroid group (although there was a slight improvement from untreated to treated, but not significant) [23]. This entertains the possibility that a longer treatment period is needed to fully see blue-yellow color contrast sensitivity return to normal or that it is permanently altered in hypothyroid populations.

A patient with hypothyroidism was the subject of a case report to assess Ricco’s area, the area of spatial summation, and its color perception [24]. It was found that the ganglion cells in Ricco’s area were temporarily dysfunctional, and after three months of treatment with levothyroxine, those cells regained normal function [24]. Following this by the same researcher, a study of 25 pretreatment hypothyroidism patients was subjected to the Farnsworth-Munsell 100 Hue test [25]. In this test, it was found that individuals with hypothyroidism have a significant deficit in their ability to distinguish color on the blue-yellow axis. In contrast, there is no deficit in distinguishing the red-green axis [25]. However, in all these studies on hypothyroidism and vision, further research is needed to be conducted due to the small sample sizes.

Effects on Thermoregulation

Finally, the effects of hypothyroidism on thermoregulation, the main symptom of this disease, will be evaluated. In a study of 42 patients with hypothyroidism before and after treatment, they were tested for thermogenesis by calorimeter in warm and mild cold stimulated rooms [26]. The patients were initially placed in a warm room at 75℉ while wearing a blanket. Then, they were exposed to a room where the temperature decreased gradually from 75℉ to 57℉ while only wearing a T-shirt and shorts [26]. The patients were then treated for hypothyroidism for three months and returned for repeat testing [26]. The results showed that upon proper treatment of hypothyroidism in these patients, there was a significant increase in their cold-induced thermogenesis [26]. This study shows that hypothyroidism affects one’s ability to regulate body temperature properly.

However, the mechanisms behind reduced thermoregulation are more complicated than one would initially think. For instance, when a hypothyroid patient is exposed to anesthesia, the hypothermia-like condition is more related to synaptic delays as opposed to thyroid hormone deficiency [27]. Other studies compared brown adipose tissue (BAT) and thyroid hormone under cold exposure. Since BAT and T3 (through conversion of T4) are both activated when the body is exposed to cold temperatures, hypothyroidism is expected to influence BAT [28]. However, this study of eight patients with thyroid cancer in both hypothyroid and thyrotoxic conditions did not show a significant short-term relationship (two to four weeks) between thyroid hormone levels and BAT mass [28]. In this specific study, it is possible that a significant relationship could come from a patient who has had hypothyroidism for a longer period, and this next study tested just that.

A study of 14 females with autoimmune hypothyroidism (ATD) was compared with 12 controls on their thermogenic response to cold temperatures [29]. In this study, the results found that the females with ATD had a reduced response to cold stimulation and yet still had increased BAT activation compared to the control group [29]. The study posits that although decreased BAT activation would normally be found in patients with hypothyroidism because of higher TSH levels, it appears to mitigate any of the ill effects that would normally affect BAT [29]. Similar research has also been conducted with hypothyroid mice and the effects of leptin on thermoregulation [30]. High leptin levels were found to be necessary for body temperature homeostasis in hypothyroid mice [30]. At room temperature, leptin was found to be elevated, but in thermoneutral conditions, leptin was not present, and hypothermia was induced [30]. Therefore, through this study, leptin has been found to have effects not previously known on the hypothalamic-pituitary-thyroid axis of thyroid hormone regulation [30]. These papers further complicate the mechanism of hypothyroidism in thermoregulation and open the door to more research that needs to be conducted on a larger scale.

## Conclusions

The relationship between hypothyroidism and olfaction is complex and not fully understood. While some studies indicate a significant impact of hypothyroidism on the sense of smell, others show no substantial difference when compared to control groups. However, there is evidence suggesting that more severe forms of hypothyroidism, particularly clinical hypothyroidism, can lead to a noticeable decline in olfactory function. Furthermore, the research indicates a significant correlation between hypothyroidism and hearing impairment, both in adults with Hashimoto's thyroiditis and children with congenital hypothyroidism. The structural changes in the inner ear's membranes are identified as a primary cause of sensorineural hearing loss, which is more prevalent in the hypothyroid group across various frequencies. Despite early and appropriate treatment for congenital hypothyroidism, children still exhibit hearing impairments, suggesting that the condition's severity may have a lasting impact on auditory function. These findings underscore the importance of monitoring auditory health in individuals with hypothyroidism and warrant further research into the mechanisms and management of associated hearing loss.

While hypothyroidism has been shown to impact several aspects of vision, including the risk of macular degeneration and visuospatial processing, certain visual functions like flicker fusion frequency remain unaffected. The condition's influence on color perception is notable, with thyroid hormone playing a crucial role in regulating color opsins. However, treatment for hypothyroidism appears to mitigate some of these effects, as evidenced by the improvement in color contrast sensitivity after treatment. This highlights the importance of ongoing research and treatment in the management of hypothyroidism's effects on vision. Finally, the relationship between hypothyroidism and thermoregulation is multifaceted and complex. While treatment for hypothyroidism shows a clear improvement in cold-induced thermogenesis, the underlying mechanisms affecting thermoregulation involve more than just thyroid hormone levels. Factors such as synaptic function during anesthesia and the role of BAT under cold exposure contribute to the overall impact of hypothyroidism on the body's ability to regulate temperature. Further research, particularly long-term studies, may provide deeper insights into these mechanisms and their interactions with thyroid health.
